# Analysis of Essential Viral Gene Functions after Highly Efficient Adenofection of Cells with Cloned Human Cytomegalovirus Genomes 

**DOI:** 10.3390/v6010354

**Published:** 2014-01-23

**Authors:** Endrit Elbasani, Ildar Gabaev, Lars Steinbrück, Martin Messerle, Eva Maria Borst

**Affiliations:** Department of Virology, Hannover Medical School, Carl-Neuberg-Strasse 1, 30625 Hannover, Germany; E-Mails: elbasani.endrit@mh-hannover.de (E.E.); gabaev.ildar@mh-hannover.de (I.G.); steinbrueck.lars@mh-hannover.de (L.S.); messerle.martin@mh-hannover.de (M.M.)

**Keywords:** human cytomegalovirus, bacterial artificial chromosomes, essential viral genes, transfection, adenovirus

## Abstract

Human cytomegalovirus (HCMV) has a large 240 kb genome that may encode more than 700 gene products with many of them remaining uncharacterized. Mutagenesis of bacterial artificial chromosome (BAC)-cloned CMV genomes has greatly facilitated the analysis of viral gene functions. However, the roles of essential proteins often remain particularly elusive because their investigation requires the cumbersome establishment of suitable complementation systems. Here, we show that HCMV genomes can be introduced into cells with unprecedented efficiency by applying a transfection protocol based on replication-defective, inactivated adenovirus particles (adenofection). Upon adenofection of several permissive cell types with HCMV genomes carrying mutations in essential genes, transfection rates of up to 60% were observed and viral proteins of all kinetic classes were found expressed. This enabled further analyses of the transfected cells by standard biochemical techniques. Remarkably, HCMV genomes lacking elements essential for viral DNA replication, such as the lytic origin of replication, still expressed several late proteins. In conclusion, adenofection allows the study of essential HCMV genes directly in BAC-transfected cells without the need for sophisticated complementation strategies.

## 1. Introduction

Human cytomegalovirus (HCMV) is still a serious threat for immunocompromised individuals such as AIDS patients and transplant recipients [1,2]. HCMV is also the leading viral cause of birth defects, resulting in severe long-term sequelae, e.g., hearing loss or mental retardation [3]. Currently, there is no effective CMV vaccine in use and the available medication has severe side effects (myelosuppression, nephrotoxicity) and can lead to the emergence of drug-resistant virus strains [4,5]. Understanding HCMV gene functions is necessary to identify new antiviral drug targets and for the rational design of a CMV vaccine. The 240-kbp HCMV genome is the largest among all mammalian DNA viruses, and according to a recent study it has the potential to encode more than 700 gene products [6]. The cloning of cytomegalovirus genomes as infectious bacterial artificial chromosomes (BAC) in *E. coli* and the establishment of suitable mutagenesis techniques has tremendously advanced the investigation into HCMV gene products [7]. Still, especially the roles of essential viral proteins remain largely elusive, which is mainly due to the lack of appropriate complementation systems. Random mutagenesis of HCMV genomes indicated that approximately 40 of the proposed 165 canonical open reading frames (ORF) [8,9] are essential for viral growth *in vitro* [10,11]. Yet, several features of HCMV, e.g., its slow replication cycle and the limited range of cell types supporting efficient growth, have impeded attempts to complement mutants with mutations in essential viral genes. Successful examples include a few cell lines expressing the essential protein of interest [12,13,14,15,16], as well as inducible systems based on tetracycline-regulated transcription [17,18], or the fusion of essential HCMV proteins to a destabilizing domain [19,20,21,22,23]. However, each of these procedures has limitations, such as low virus productivity on the complementing cells, occurrence of escape mutants (rescuants), insufficient tightness of conditional gene regulation, or impairment of viral protein function upon fusion to regulatory domains. 

We therefore asked whether it is possible to analyze the phenotypic consequences of the disruption of an essential viral ORF directly in cells transfected with the respective mutant HCMV BACs. Transfection of permissive primary cells with the 240-kbp HCMV BACs is very inefficient, resulting in few transfected cells only, and immortalized cell lines widely employed for complementation of α-herpesvirus mutants do not support the full replication cycle of HCMV. Utilizing commercially available transfection reagents, infectious viral progeny can be reconstituted from HCMV BACs, because few successfully transfected cells will give rise to infectious progeny that finally spreads throughout the cultures. However, the transfection efficiencies achieved so far did not allow the analysis of the transfected cells by common virological and biochemical techniques. In this study, we evaluated an alternative method to introduce HCMV BACs into permissive cells, namely an adenovirus-based gene delivery protocol that was pioneered by Matthew Cotten and coworkers [24,25,26,27]. Adenovirus particles derived from a replication-deficient mutant are chemically and physically inactivated and serve as carriers for the HCMV BACs, which results in the uptake of the BACs via the natural infection route of the adenovirus. Firstly, the BAC DNA is condensed with cationic low molecular weight polyethyleneimine (PEI), yielding a BAC-PEI complex with an overall positive charge. Secondly, this complex is bound to the adenovirus (Ad) particles, presumably through interaction with the negatively charged hexon protein of the viral capsid. By receptor-mediated endocytosis, the BAC-PEI-Ad complexes are then taken up by the cells, and the ability of PEI to act as a proton sponge together with the endosomolytic activity of adenovirus capsid proteins enable the escape of the transfected DNA into the cytosol. 

We applied this method, which we termed adenofection, to different HCMV-permissive cell types and used various HCMV BACs in which essential genomic regions had been deleted. The excellent delivery rates of the mutated HCMV genomes, particularly into an epithelial cell line, made it possible to examine the transfected cells for expression, localization, and interactions of viral proteins, as well as for testing of viral DNA replication. In summary, by using the adenofection technique, essential viral gene functions can be assessed immediately after transfection of the HCMV BAC mutants, which obviates the requirement to develop individual complementation strategies.

## 2. Results and Discussion

### 2.1. Adenovirus-Mediated Delivery of HCMV BACs into Different Cell Types and Optimization of the Procedure

**Figure 1:**
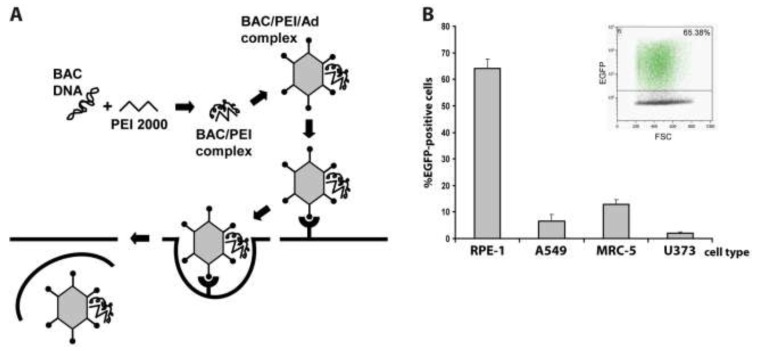
(**A**) Principle of the adenofection method. BAC DNA is condensed with low molecular weight polyethylenimine (PEI 2000). Addition of replication-incompetent, chemically and physically inactivated adenovirus particles leads to binding of the BAC-PEI complexes to the virions. The DNA-loaded adenovirus particles are taken up by the cells, and the endosomolytic activity of virion protein helps to release the DNA into the cytosol; (**B**) Adenofection of different human cell types with the EGFP-expressing HCMV BAC pHG-13. RPE-1: retinal pigmented epithelial cell line, A549: lung adenocarcinoma epithelial cell line, MRC-5: primary embryonic lung fibroblasts, U373: human astrocytoma cell line. Transfection efficiency is given as percentage of EGFP-positive cells on day 1 post transfection. Transfections were done in quadruplicates, and standard deviations are indicated. Inlay: example of FACS analysis of adenofected RPE-1 cells.

Previously, we had already set up the adenofection method (Figure 1A) to reconstitute virus from HCMV BACs in primary human foreskin fibroblasts (HFF) [28]. HFF is the prototype cell type for propagating HCMV, however, these primary cells are particularly difficult to transfect. In these previous experiments, adenofection gave better results than all commercially available transfection reagents we had tried before, yielding approximately 100–200 transfected cells per 3.5 cm dish. Here, we examined the transfection efficiency of other human cell types after adenofection with an EGFP-expressing HCMV BAC (pHG-13) that does not give rise to infectious progeny due to a deletion in the essential ORF UL104 (see Table 1). Since primary HFF originate from various sources and may therefore vary in their properties, we instead used commercially available primary fetal lung fibroblasts (MRC-5), which in addition are also easier to transfect than HFF [29]. Besides MRC-5, we tested two epithelial cell lines, retinal pigmented epithelial cells having an extended life span due to expression of human telomerase reverse transcriptase (hTERT-RPE-1 cells, further referred to as RPE‑1), and A549 cells (a lung adenocarcinoma epithelial cell line) as well as the human glioblastoma cell line U373. All of these cells can be infected with HCMV and support replication of the virus, although with different efficacy. Because the adenofection protocol is sensitive to bacterial lipopolysaccharide (LPS) that is potentially present in plasmid preparations and interferes with DNA‑PEI complex formation and can also provoke cytotoxicity in the presence of adenovirus [30], we first compared BAC DNA purified by standard protocols to that obtained by a method removing most of the LPS. Indeed, when using endotoxin-free BAC DNA, transfection rates increased twofold over those obtained with LPS-containing DNA (data not shown), even though the LPS-chelating substance polymyxin B was added during adenofection, which alleviates LPS-mediated toxicity [31]. Therefore, all of the following experiments were done with endotoxin-free BAC DNA. The different cell types were adenofected with 1 µg of pHG-13 each, and EGFP expression was analyzed the following day by flow cytometry. As can be seen in Figure 1B, transfection of RPE-1 cells was most efficient, with 64% of cells scoring positive for EGFP, followed by MRC-5 (13%), A549 (6.5%) and U373 cells (2%). To our knowledge, such high delivery rates of HCMV BACs into permissive cells have not been achieved previously by applying any other transfection technique. It is conceivable that by optimizing parameters even higher transfection efficiencies can be achieved. It has for instance been reported that the success of the method greatly depends on the formation of a DNA-PEI complex which is of optimal size and has a positive net charge [27]. The size of these complexes can be tuned by varying the sodium chloride concentration of the HBS solution employed to dilute both DNA and PEI (see Experimental Section), and the charge of the complex is given by the molar ratio of the anionic DNA to polyethylenimine (a polycation). We performed all adenofection experiments in the presence of 150 mM sodium chloride as initially proposed [24,26], which results in larger complexes compared to that prepared in the absence of sodium chloride [27]. If smaller complexes are beneficial to transfection of HCMV BACs remains to be tested. Related to that, we noticed that the transfection of HCMV BACs is enhanced when increasing the amount of PEI 2000. For smaller DNA molecules, maximum delivery was reported when the molar ratio of PEI nitrogen to DNA phosphate (N:P) was around 10, and was not further improved using higher N:P ratios [27]. However, there is some evidence that larger DNA molecules may require a higher N:P ratio than smaller plasmids [26]. In our HCMV BAC transfection protocol the N:P ratio is 50 (1 µg of DNA equals 3 nmol of phosphate, and 15 µL of 10 mM PEI 2000 solution correspond to 150 nmol of nitrogen). We also tried N:P ratios of 33 and 67 (see below) and did not see a major effect on transfection efficiency, yet lowering the N:P ratio to 20 resulted in a markedly decreased delivery rate (not shown). Therefore, by determining the best relation between sodium chloride, HCMV BAC DNA, PEI 2000, and also the amount of adenovirus particles (*i.e.*, the size and charge of the final PEI-DNA-adenovirus complex that is taken up by the cells) the procedure may be further improved.

**Figure 2:**
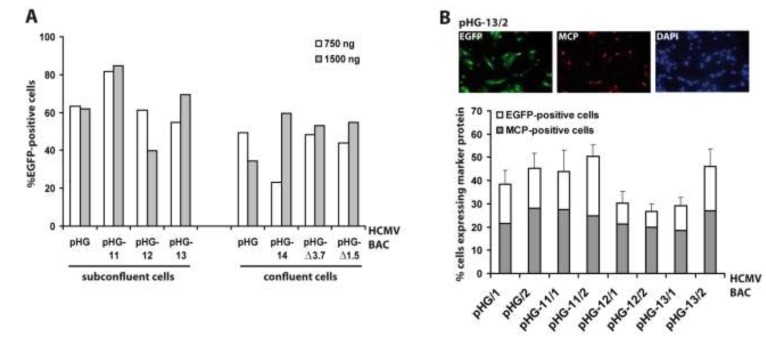
Evaluation of adenofection conditions and late protein expression in adenofected cells. (**A**) Subconfluent or confluent RPE-1 cells were transfected with 750 or 1,500 ng of the HCMV BACs indicated. On day 1 post transfection the percentage of EGFP-expressing cells was determined by flow cytometry. Each bar represents the mean value of two transfections; (**B**) RPE-1 cells transfected with the HCMV BACs indicated were stained on day 4 post transfection with DAPI and an antibody against the major capsid protein (MCP). Ten random images were taken per setting, and the percentages of EGFP-positive cells (white bars) and MCP-expressing cells (grey bars) were determined. The index numbers following the names of the BACs (/1 and /2) indicate that 750 and 1,500 ng of BAC DNA were used for adenofection, respectively.

Since RPE-1 cells were most efficiently transfected by adenofection, all subsequent experiments were performed with this cell type. Next we tested whether the amount of BAC DNA and the cell confluence have an impact on transfection efficiency. Six different HCMV BACs harboring deletions in essential genomic regions (pHG-11 to pHG-14, pHG-Δ3.7, and pHG-Δ1.5; see Table 1) and the parental BAC pHG were used to transfect either 80% or 100% confluent RPE-1 cells with 0.75 µg (N:P = 67) or 1.5 µg (N:P = 33) of each BAC DNA, respectively (Figure 2A). One day post transfection, the number of EGFP-expressing cells was determined by flow cytometry. The DNA amounts used had little influence on the number of EGFP-positive cells, whereas (as also true for other transfection protocols) there was a slight improvement in transfection efficiency when using subconfluent cell cultures. Occasionally we observed variable transfection efficiencies (e.g., compare pHG-14 to pHG, white bars in Figure 2A, right part), which is perhaps due to the fact that preparations of high molecular weight DNA such as of the 240 kb HCMV BACs tend to be inhomogeneous, resulting in differing DNA contents during handling of the samples. Still, in general adenofection allowed the uptake of the HCMV BACs into 40%–60% of the RPE-1 cells, which represents an outstanding delivery rate not obtained with any other transfection reagent tested so far [32]. 

**Table 1:** HCMV BACs used in this study.

Name of HCMV BAC	Mutation
pHG (parental BAC)	EGFP ORF under control of HCMV MIEP deletion of UL1-10 [28]
pHG-11	deletion in UL77
pHG-12	deletion in UL93
pHG-13	deletion in UL104
pHG-14	deletion in UL44
pHG-Δ1.5	1.5 kb deletion in oriLyt [28]
pHG-Δ3.7	3.7 kb deletion in oriLyt [28]
pHG-Δ52	deletion in UL52 [14]

### 2.2. Viral Gene Expression in Adenofected Cells

So far, we measured the outcome of adenofection by detection of EGFP, which is under control of the HCMV major immediate-early promoter (MIEP) [33]. EGFP-expression thus directly represents transfection efficiency, because gene expression driven by the HCMV MIEP occurs very early after transfection (and infection) and is independent of the synthesis of other viral proteins. However, most experiments to be performed with HCMV BAC-transfected cells will not only require the initiation of viral gene expression, but also progression to the early and late phases of the HCMV life cycle. Therefore, we analyzed how many of the EGFP-positive cells proceed to the late phase of the infection cycle. A portion of the cells transfected with pHG or pHG-11 to pHG-13 (*cf.* Figure 2A, left part) was seeded onto cover slips and examined on day four post transfection for the presence of EGFP and the major capsid protein (MCP; a late viral protein) by immunofluorescence (Figure 2B). Compared to the data obtained by FACS analysis, transfection efficiency evaluated by fluorescence microscopy gave slightly lower values, probably because the latter technique is the less sensitive one. We found that in the majority of the EGFP-expressing cells MCP was detected as well, and also displayed the expected nuclear localization, with the percentage of MCP-positive cells (related to the EGFP-expressing cells) ranging from 50% (pHG-11/2) to 75% (pHG-12/2). This result is similar to what is observed after infection with HCMV, where it is known that not all cells expressing immediate-early proteins also make it into the late phase and lead to plaque formation, which can be explained by defective virus particles and/or an unfavorable cellular environment (e.g., failure of viral genomes to overcome intrinsic cellular defense mechanisms such as sequestration at PML bodies). Likewise, it is conceivable that not all of the 240 kb HCMV BAC molecules remain intact during the DNA preparation and transfection procedure. Moreover, progression through the productive infection cycle may be impaired in the absence of tegument proteins that usually counteract the function of cellular restriction factors early in infection [34,35]. This may be overcome by simultaneously transfecting the cells with plasmids encoding the respective tegument proteins, such as pp71 which is known to enhance gene expression from HCMV DNA [36].

**Figure 3:**
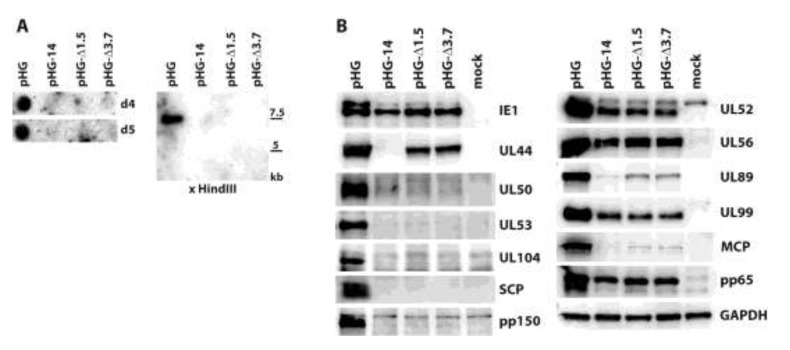
Analysis of viral genome replication and protein expression in adenofected cells. (**A**) Viral DNA levels in RPE-1 cells adenofected with the indicated HCMV BACs. Total DNA was isolated on days four and five post transfection and analyzed by dot blot hybridization using a UL93-specific probe (left part). Right part: the DNA samples obtained on day five were treated with HindIII and DpnI, followed by Southern blotting. The ^32^P-labelled probe specific for the UL93 ORF detects a 7.5 kb HindIII fragment; (**B**) Viral protein expression after adenofection with the depicted HCMV BACs. Cell lysates were prepared on day four post transfection and analyzed for the given proteins by immunoblotting.

Next, we aimed at detecting expression of other viral proteins following adenofection and also included early and additional late proteins. Furthermore, we wanted to investigate late gene expression in the absence of HCMV genome replication. The kinetics of herpesviral protein expression is typically analyzed by keeping infected cells in the presence of inhibitors that interfere with protein synthesis (e.g., cycloheximide, CHX) or DNA replication (phosphonoacetic acid, PAA). Viral genes transcribed in the presence of CHX are classified as immediate-early, and those expressed despite the presence of PAA are considered to be early genes. In contrast, it is believed that late proteins are synthesized only in the absence of any inhibitor, *i.e.*, following viral genome replication. Cells were adenofected with HCMV BACs pHG-14, pHG-Δ1.5 or pHG-Δ3.7 to investigate whether late protein synthesis strictly relies on viral replication. pHG-14 has a deletion in the UL44 open reading frame (ORF), whose gene product pUL44 is the accessory factor of the viral DNA polymerase and is required for HCMV lytic origin (oriLyt)-dependent genome replication [37,38]. In pHG-Δ1.5 and pHG-Δ3.7, a 1.5 kb or 3.7 kb region of oriLyt was deleted, and upon transfection these BACs do not produce infectious progeny [28]. We first ensured that these BACs have indeed a defect in viral genome replication. Adenofected RPE-1 cells were harvested on days 4 and 5 post transfection, and total DNA was extracted from the cells. The DNA samples were examined by dot blot as well as by restriction analysis, followed by Southern blotting. As is obvious from Figure 3A, viral DNA was only detected in cells transfected with the parental BAC pHG, but not after transfection of pHG-14, pHG-Δ1.5, or pHG-Δ3.7. We conclude from this data that these HCMV genomes are deficient for viral DNA replication.

We then checked viral protein expression in the adenofected cells by immunoblotting (Figure 3B). The 72 kDa IE1 protein (Figure 3B, panel IE1, lower band) was detected in comparable amounts with all of the mutants as well as with the parental BAC. Interestingly, the higher molecular mass form of IE1, presumably representing sumoylated IE1, was markedly reduced when viral replication was impaired, in line with observations of others which showed that this version of IE1 is rather made during the late phase of the HCMV life cycle [39]. UL44, an early gene product, was expressed by all BACs except pHG-14, in which the UL44 ORF was disrupted. Remarkably, compromising viral replication had different effects on the levels of the other proteins tested, ranging from almost complete absence (UL50, UL53, UL104, SCP, pp150: all late proteins) to more or less reduced, but still clearly detectable amounts (UL52, UL99, MCP, pp65: late proteins, and UL56, UL89: early-late proteins). It is also of note that UL52, which was previously designated as a true late protein due to its complete lack of expression in the presence of PAA [14,40] was found in considerable amounts after transfection of the replication-defective genomes. These data may be explained by recent results of Nitzsche and colleagues, who found that viral DNA replication is needed to mark the HCMV genome with methylated histones that define transcriptionally active chromatin, namely H3K4me2 [23]. Most interestingly, the tested HCMV genomic regions were differentially influenced by impairing viral replication, for instance occupancy of UL32 sequences (coding for pp150) with H3K4me2 was much more dependent on HCMV genome replication than that of the UL99 gene, which was still labeled with this activating histone mark quite efficiently in both the presence of ganciclovir and after knock-down of the viral polymerase. This is consistent with the expression of the UL99 protein and the absence of pp150 (UL32) in cells transfected with HCMV BACs defective for genome replication. A similar mechanism may apply to the other HCMV proteins tested by us in this study. 

### 2.3. Impact of UL52 on HCMV Terminase Proteins

Besides genome replication, the nuclear phase of the HCMV life cycle comprises the assembly of capsid shells, as well as packaging of viral genomes into these preformed capsids. HCMV proteins mediating genome cleavage and encapsidation are the terminase subunits UL56 and UL89. We have recently demonstrated that UL51 is also essential for cleavage of viral concatemeric DNA and the generation of DNA-filled capsids. Moreover, UL51 forms a complex with UL56 and UL89 and seems to be required for the correct localization of UL56 and UL89 to nuclear replication compartments [40,41]. Likewise, we have identified an essential role of UL52 in genome cleavage-packaging, but there was no evidence for UL52 being also part of the terminase complex [14,40]. The precise function of UL52 thus remains elusive.

**Figure 4:**
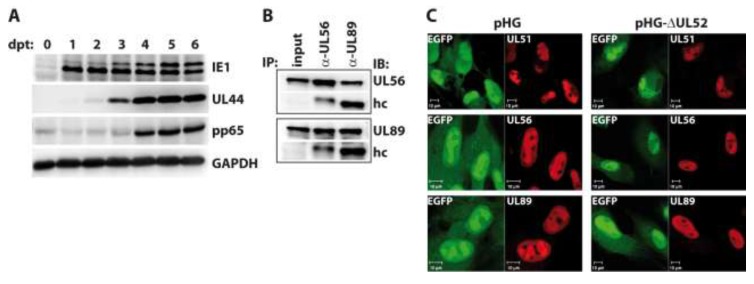
Investigation into RPE-1 cells adenofected with the UL52-deleted genome pHG-Δ52. (**A**) Transfected cells were harvested on days zero to six post transfection (dpt) and expression of the indicated proteins was analyzed by immunoblotting. Please note that the pp65 antibody exhibits some cross-reactivity with cellular proteins as observed previously [42]; (**B**) Interaction of the terminase subunits UL56 and UL89 in the absence of UL52. RPE-1 cells adenofected with pHG-Δ52 were subjected to immunoprecipitation with α-UL56 and α-UL89 monoclonal antibodies on day 4 post transfection, followed by immunoblotting using the same antibodies. Input: whole cell lysate before IP, hc: IgG heavy chain; (**C**) Subcellular localization of HCMV terminase subunits in the presence or absence of UL52. RPE-1 cells were adenofected with pHG or pHG-Δ52 and analyzed on day 4 post transfection by confocal laser scanning microscopy using monoclonal antibodies against UL51, UL56, and UL89.

We now asked whether the absence of UL52 may influence the interactions among the terminase subunits or their subnuclear localization. To this end, RPE-1 cells were adenofected with the BAC genome pHG-ΔUL52, which carries a disrupted UL52 ORF. First, we checked the expression kinetics of representative immediate-early (IE1), early (UL44) and late proteins (pp65) in the transfected cells. Cells were harvested on days 0 to 6 post transfection, and viral proteins were analyzed by immunoblotting. As is obvious from Figure 4A, IE1, UL44 and pp65 were expressed with the expected kinetics, with IE1 already being detectable on day 1, UL44 coming up on day 2, and pp65 being clearly visible on day 4 and later. The higher molecular mass form of IE1 was detected in distinct amounts on day 3 and later only, which is in agreement with our finding that the abundance of this version of IE1 seems to be increased following viral replication (*cf.* Figure 3B). To investigate the interaction of the terminase subunits, pHG-ΔUL52-transfected cells were lysed on day four and subjected to immunoprecipitation using monoclonal antibodies (mAb) directed against UL56 and UL89 [40]. Figure 4B shows that the binding of UL56 to UL89 was still seen when UL52 was missing, since immunoprecipitation of UL56 also brought down UL89 (Figure 4B, second lane), and vice versa (Figure 4B, third lane). We could not test for binding to UL51 in this setting, because the UL51 mAb exhibits no distinct reactivity in immunoblotting. Since the UL51 antibody performs well in immunofluorescence experiments, we examined the subcellular distribution of UL51, UL56 and UL89 in the absence of UL52 by confocal laser scanning microscopy in RPE-1 cells transfected with pHG-ΔUL52 or the parental BAC pHG. As can be seen in Figure 4C, the terminase subunits were all found in the cell nucleus and were enriched within the replication compartments, despite the lack of UL52 (Figure 4C, right part), as is the case in cells transfected with the parental BAC genome pHG (Figure 4C, left part). Thus, UL52 does not seem to play a role in the interactions among the HCMV terminase proteins or their correct intracellular localization.

## 3. Experimental Section

### 3.1. Cells

hTERT-RPE-1 cells were obtained from Clontech and were cultivated in Dulbecco’s modified Eagle’s medium (DMEM)/Ham’s F-12 (Sigma, Saint Louis, MO, USA, D6421), supplemented with 5% fetal calf serum (FCS), 100 U/mL of penicillin, 100 µg/mL of streptomycin sulfate, 2 mM glutamine, and 0.348% sodium bicarbonate. A549 and MRC-5 cells (ATCC) were kept in DMEM (Sigma, D5796), containing 10% FCS and the mentioned antibiotics. The U-373 MG (Uppsala) cell line was purchased from Sigma (Cat. No. 08061901) and corresponds to a new deposit of the cells from the laboratory in Uppsala, Sweden, where they were originally isolated. U-373 MG cells were cultivated according to the instructions of the supplier. 

### 3.2. HCMV Bacterial Artificial Chromosomes

The parental HCMV BAC pHG, carrying the enhanced green fluorescent protein (EGFP) ORF under control of the major immediate-early promoter (MIEP) and containing a deletion of the non-essential genes UL1 to UL10 was described before [28,33]. The construction of pHG-Δ52 lacking most of the UL52 open reading frame (ORF) was reported elsewhere [14], and pHG-Δ1.5 and pHG-Δ3.7 harboring deletions in the HCMV lytic origin region have also been described [28]. pHG-11, pHG-13 and pHG-14, having deletions in essential viral genes UL77, UL104 and UL44, respectively, were generated by red αβγ-mediated recombination in *E. coli* as described [43]. Oligonucleotides used for mutagenesis are given in Table 2, and comprised sequences homologous to the desired integration site in the BAC, as well as priming regions to the kanamycin resistance (KnR) gene flanked by mutant FRT sites [28]. The KnR sequence was subsequently removed from all BACs by Flp-mediated recombination as reported [43]. pHG-12, in which UL93 is disrupted, was constructed by *en passant* mutagenesis using the primers indicated in Table 2, with a KnR cassette carrying an I-SceI site as a template [44]. Further details on the construction of these BACs will be available from the authors upon request. Large-scale purification of HCMV BACs was done either with the Nucleobond PC 100 columns, or by using the Nucleobond PC 500 EF kit (both Macherey & Nagel, Düren, Germany), which yields mostly endotoxin-free DNA. BAC DNA was resuspended in TE buffer (10 mM Tris-HCl pH 8.0/1 mM EDTA) and DNA concentrations were determined by measuring each preparation three times using a Nanodrop device.

**Table 2:** Oligonucleotides used for HCMV BAC mutagenesis.

Primer name	Nucleotide sequence
UL77-ko.for	5'-acgatgccatcacgggacccgccgccgccccgtctgacgtggaaaagtgccacctgcagat-3'
UL77-ko.rev	5'-ccgaggacgttcgccctttatgcagcgagcgacacgtggtgcaggaacacttaacggctga-3'
UL104-ko.for	5'-gctgtcgtcgtaggcggcggccacgatctcgccgaaggagagaaaagtgccacctgcagat-3'
UL104-ko.rev	5'-gtgctgggcggcctccgcgacattttatatcagtacgccgcaggaacacttaacggctga-3'
UL44-ko.for	5'-gggaccgcgccgtgcgcgcgttcccaggcacgcggcccgcgaaaagtgccacctgcagat-3'
UL44-ko.rev	5'-tcgctcgctcgcgcccgctccttagtcgagacttgcacgctcaggaacacttaacggctga-3'
UL93-ko.for	5'-agtcgaactataccgggcgttggacgcttatcgggcgtagtgactgattcgcgccgtgcgcaa-ggatgacgacgataag-3'
UL93-ko.rev	5'-agtacagcgtagatctcgtcgcgcacggcgcgaatcagtcactacgcccgataagcgtccagcc-agtgttacaa­ccaatt-3'

### 3.3. Preparation of Adenovirus Stocks and Transfection by Adenofection

The adenovirus dl1014 mutant [45], having a deletion in the E4 region, and the complementing cell line W162 [46] were kindly provided by Gary Ketner, Johns Hopkins University School of Medicine, MD, USA. To produce the adenovirus stock, ten T175 flasks of W162 cells were infected at an MOI of 0.01–0.1. At approximately day three post infection, when all cells were rounded and detached, cells were collected by centrifugation and the pellet was transferred to −80 °C. Virus was released by adding 5 mL of serum-free DMEM, followed by two freeze-thaw cycles. Cellular debris was removed by centrifugation at 3,800 × g for 20 min, and virus in the supernatant was purified by two rounds of isopycnic cesium chloride gradient centrifugation. For the first centrifugation step, 3 mL of a CsCl solution with a density of 1.2 g/mL was carefully added to a tube containing 3 mL of a CsCl solution with a density of 1.4 g/mL. Virus-containing supernatant was layered on top, and centrifugation was done for 90 min at 100,000 × g, 20 °C in an SW41 Ti rotor, without deceleration. The virus-containing band was identified by light scattering and removed by puncturing the tube with a hollow needle, and was then diluted to a total volume of 2 mL with 10 mM Tris-HCl/1 mM EDTA pH 7.5. The second purification step was done via a continuous CsCl gradient prepared by mixing 5.4 mL of CsCl solution density 1.2 with 4.6 mL of CsCl solution density 1.4 using a gradient maker, and centrifugation took place for 24 h at 100,000 × g, 20 °C without deceleration. After harvesting of the virus band as described above, the samples were desalted using PD-10 columns (GE Healthcare, Uppsala, Sweden), and fractions of 0.5 mL each were collected by elution in HBS/40% glycerol (HBS is 20 mM HEPES, 150 mM NaCl pH 7.4). Virus-containing fractions were identified by measuring protein concentrations with the Pierce BCA protein assay kit (Thermo Scientific, Rockford, IL, USA), and protein-positive fractions were pooled. To inactivate the virus, 8-methoxy-psoralen (Sigma, M-3501) was added to a final concentration of 0.33 mg/mL, the sample was transferred on ice and exposed to UV light (366 nm) for 30 min, with rotation of the dish every 10 min. The inactivated virus particles were again purified by PD-10 columns, and fractions scoring positive in the BCA protein assay were pooled and stored at −80 °C in HBS/40% glycerol. Concentration of adenovirus particles was determined by calculating 0.3 mg/mL of (capsid) protein being equivalent to 1 × 10^12^ particles/mL.

For adenofection of HCMV BACs, the DNA amounts indicated in the figures were diluted in 250 µL of HBS. 15 µL of 10 mM PEI 2000 stock solution (polyethylenimine MW 2000, Sigma, Cat. No. 40870-0, 0.9 mg/mL H_2_O), mixed before usage, was diluted in 250 µL of HBS and mixed vigorously again. The diluted PEI 2000 solution was slowly added to the diluted DNA, with constant gentle flicking of the tube, and the sample was incubated for 20 min at room temperature (RT). 3 × 10^9^particles of inactivated adenovirus were added, and the sample was incubated at RT for another 20 min. Cells cultured in six-well plates were washed with PBS, and 1.5 mL/well of serum-free DMEM containing polymyxin B (30 µg/mL) was added. The transfection complexes were added to the cells, which were then incubated for 5 h at 37 °C, 5% CO_2_. After that, the cells were washed twice with PBS and further cultivated in complete growth medium with polymyxin B (30 µg/mL). 

### 3.4. Flow Cytometry

Cells were adenofected with the HCMV BAC genomes indicated in the figure legends. One day later, cells were trypsinized, washed with PBS containing 2 mM EDTA, and fixed with 3% paraformaldehyde (*w*/*v*) in PBS/2mM EDTA. One-color flow cytometry was applied to detect GFP-positive cells. A total of 30,000 events were collected by a Beckman Coulter FC500 flow cytometer and analyzed using the Kaluza software (Beckman Coulter, Brea, CA, USA) [47]. 

### 3.5. Viral DNA Replication Assay

RPE-1 cells adenofected with the respective HCMV BACs were trypsinized on days four or five post transfection, and total DNA was isolated using the DNeasy Blood and Tissue kit (Qiagen, Hilden, Germany). For dot blot, 5 µL of each sample (corresponding to approximately 30 ng of total DNA each) was applied to a nylon membrane. Denaturing of the DNAs was achieved by exposing the membrane to filter paper soaked with 0.5 M NaOH/1.5 M NaCl twice for three min each, followed by neutralization of the membrane four times for three min each using 0.5 M Tris-HCl/1.5 M NaCl pH 7.2. For restriction analysis, 40 µL of each DNA sample obtained on day five (corresponding to approximately 240 ng of total DNA each) was treated with DpnI to cut residual transfected BAC DNA, followed by treatment with HindIII. After gel electrophoresis, the DNAs were transferred to a nylon membrane by Southern blotting, and hybridized to a ^32^P-labelled probe generated by PCR amplification of HCMV UL93 sequences using primers UL93-N.for (5'-cgcggatccttctatgccgtcttcactacg-3') and UL93-N.rev (5'-cgcaagcttgcgactgcgccaaaaggaatt-3') with HCMV BAC pHG as a template.

### 3.6. Protein Biochemistry

Immunofluorescence microscopy of adenofected cells was done as described previously [14], and immunoprecipitation as well as immunoblotting was performed as reported recently [40]. Additionally, the SuperSignal Western Blot Enhancer reagents (Thermo Scientific, Cat. No. 46640) and the SuperSignal West Femto Maximum Sensitivity Substrate (Thermo Scientific, Cat. No. 34096) were applied. Antibodies commercially available were directed against HCMV IE1 (NEA-9221, PerkinElmer, Boston, MA, USA), UL99 (Fitzgerald, MA, USA, Cat. No. 10-C50), and pp65 (Novus Biologicals, Littleton, CO, USA, Cat. No. NB110-57244). The antibody recognizing the major capsid protein was kindly provided by Klaus Radsak, UL44 and pp150 monoclonal antibodies were a gift of Bodo Plachter, University of Mainz, Germany, UL50 and UL53 antibodies were obtained from Manfred Marschall, University of Erlangen, Germany, and the monoclonal antibody directed against the HCMV small capsid protein was provided by William Britt, Birmingham, Alabama. The monoclonal antibodies specific for UL51, UL52, UL56, and UL89 are described elsewhere [40]. The UL104-specific antibody was generated by immunization of mice as described [40], using a recombinant protein comprising the N-terminal part of pUL104. Primers for amplification of UL104 sequences were UL104-N.for (5'-cgcaagcttggcgtactgatataaaatgt-3') and UL104-N.rev (5'-cgcggatccgagcgaaaccactggaacga-3'). The resulting PCR product was cut with HindIII and BamHI and cloned into the bacterial expression vector pQE-30 via the same restriction sites. 

## 4. Conclusions

In this work, we present a novel approach to analyze essential HCMV gene functions by transfecting cells with BACs that are deleted for the gene of interest. Transfection is based on applying replication-deficient, inactivated adenovirus particles as carriers, a method that was initially developed by Cotten and coworkers for BACs of up to 170 kb, and that proved to be superior to commercially available transfection reagents [26,27]. We now show that this approach works also perfectly well with HCMV BACs, which are of larger size (240 kbp). We used particles of an adenovirus mutant that has a deletion in the essential E4 region and is propagated on a complementing cell line. However, other adenovirus mutants may be employed as well, such as dl312 lacking E1a sequences and which can be grown on 293 cells [24], although under certain conditions cellular factors can substitute for E1a functions, resulting in toxicity due to low level adenoviral gene expression [25]. Due to the natural tropism of human adenovirus the adenofection method is currently restricted to human cells. However, it is conceivable that the protocol can be extended to BAC-cloned herpesviruses of other species, for instance mouse adenovirus mutants could be generated and tested for adenofection of murine cells. Transfection can also be performed through double condensation of BAC DNA with low and high molecular weight PEI only [48]. In our hands, this approach also gave good transfection efficiencies (data not shown), although the high transfection rates described here (40%–60%) were only reached upon including adenovirus particles. 

The excellent transfection efficiencies with HCMV BACs found after adenofection have not been possible before with other transfection methods applied so far. RPE-1 cells performed best in these experiments, which is of note because this cell type is also useful to propagate clinical HCMV isolates [49]. This enables faster reconstitution of viral progeny from HCMV BAC genomes, which could be of advantage for high throughput analysis of a library of HCMV mutants. Furthermore, the new delivery approach allows the study of the phenotypic consequences of the lack of given essential proteins by various techniques such as immunofluorescence, immunoblotting, or immunoprecipitation. Other assays, for instance pulsed field gel electrophoresis of viral DNA or electron microscopy, are also feasible, especially when the adenofected cells are sorted for EGFP-positive cells before applying the respective techniques. We think that virtually any investigation can be done with BAC-transfected cells, at least as steps of the infection cycle subsequent to delivery of the viral genomes into the nucleus are concerned. Although RNAs and virion proteins delivered by the HCMV particle, which can enhance viral gene expression are absent following adenofection, all viral proteins tested were produced in sufficient amounts to perform downstream analyses. We consider the adenofection procedure as safe since infectious adenovirus was not observed in the transfection experiments. Moreover, recombination between adenoviral and HCMV DNA appears highly unlikely, because (i) the genomes do not share significant homologous regions; (ii) the adenovirus DNA is cross-linked by treatment with psoralen and UV light and is thus inert; and (iii) there is no selection pressure on the occurrence of recombination.

In summary, adenofection of HCMV BACs enables the analysis of essential viral genes directly after transfection, without the need to establish complementing systems for the HCMV mutants of interest. 
